# *PPD ACT:* an app-based genetic study of postpartum depression

**DOI:** 10.1038/s41398-018-0305-5

**Published:** 2018-11-29

**Authors:** Jerry Guintivano, Holly Krohn, Carol Lewis, Enda M. Byrne, Anjali K. Henders, Alexander Ploner, Katherine Kirk, Nicholas G. Martin, Jeannette Milgrom, Naomi R. Wray, Patrick F. Sullivan, Samantha Meltzer-Brody

**Affiliations:** 1 Department of Psychiatry, University of North Carolina at Chapel Hill, Chapel Hill, NC USA; 2 Center for Health Innovation, University of North Carolina at Chapel Hill, Chapel Hill, NC USA; 3 Institute for Molecular Bioscience, The University of Queensland, Brisbane, QLD Australia; 4 Department of Medical Epidemiology and Biostatistics, Karolinska Institutet, Stockholm, Sweden; 5 QIMR Berghofer Institute for Medical Research, Brisbane, QLD Australia; 6 Parent-Infant Research Institute and University of Melbourne, Melbourne, VIC Australia; 7 Queensland Brain Institute, The University of Queensland, Brisbane, QLD Australia; 8 Department of Genetics, University of North Carolina at Chapel Hill, Chapel Hill, NC USA

## Abstract

Postpartum depression (PPD) is one of the most frequent complications of childbirth and particularly is suited to genetic investigation as it is more homogenous than major depression outside of the perinatal period. We developed an iOS app (*PPD ACT*) to recruit, consent, screen, and enable DNA collection from women with a lifetime history of PPD to sufficiently power genome-wide association studies. In 1 year, we recruited 7344 women with a history of PPD and have biobanked 2946 DNA samples from the US. This sample of PPD cases was notably severely affected and within 2 years of their worst episode of PPD. Clinical validation was performed within a hospital setting on a subset of participants and recall validity assessed 6–9 months after initial assessment to ensure reliability of screening tools. Here we detail the creation of the *PPD ACT* mobile app including design, ethical, security, and deployment considerations. We emphasize the importance of multidisciplinary collaboration to correctly implement such a research project. Additionally, we describe our ability to customize the *PPD ACT* platform to deploy internationally in order to collect a global sample of women with PPD.

## Introduction

Approximately one in seven women experience postpartum depression (PPD) following the birth of a child. In the US, more than 500,000 women each year will suffer with PPD (lifetime prevalence of 10–15%)^1,2^ and it is one of the most frequent complications of childbirth. Prominent consequences of PPD include maternal suicide, infanticide, and reduced maternal sensitivity, which can adversely affect emotional regulation and infant attachment leading to adverse neurodevelopmental outcomes for the child^1–7^. However, despite the potentially profound effects on mother and child, PPD is often under-diagnosed, understudied, and inadequately treated^8^.

PPD is a form of major depressive disorder (MDD). PPD has an important genetic component and its heritability (44–54%) is greater than that of MDD (32%)^9,10^. PPD is particularly suited to genetic investigation as it is more homogenous than MDD: PPD affects women within childbearing years following exposure to the biopsychosocial stressors of pregnancy and childbirth^1,2,11^. The Psychiatric Genomics Consortium (PGC) recently identified 44 risk loci associated with MDD in a meta-analysis of 135,458 cases and 344,901 controls^12^, demonstrating the success of genomics for a complex psychiatric phenotype closely related to PPD. However, this work also illustrates that success requires large sample sizes collected through an international consortium to sufficiently power genome-wide association studies (GWAS).

Ascertainment, consent, and phenotyping in a psychiatric genetics study has classically involved an in-person standardized diagnostic interview to establish case–control status, typically conducted in a clinical setting. The throughput of this approach is low and limited by cost and procedural complexity. However, the recent boom of smartphones and social media offers the possibility to dramatically transform and expand our ability to engage study participants. In 2017, 75% of all women use smartphones in the US, indicating that a mobile health platform has enormous potential to bring research directly to participants. Combined with the greater acceptability of web-based mental health screening compared with paper-based screening among women^13^, app-based research can allow detailed longitudinal phenotyping on a large scale with a convenient mechanism for follow-up that would otherwise be infeasible or impossible.

**Fig. 1:**
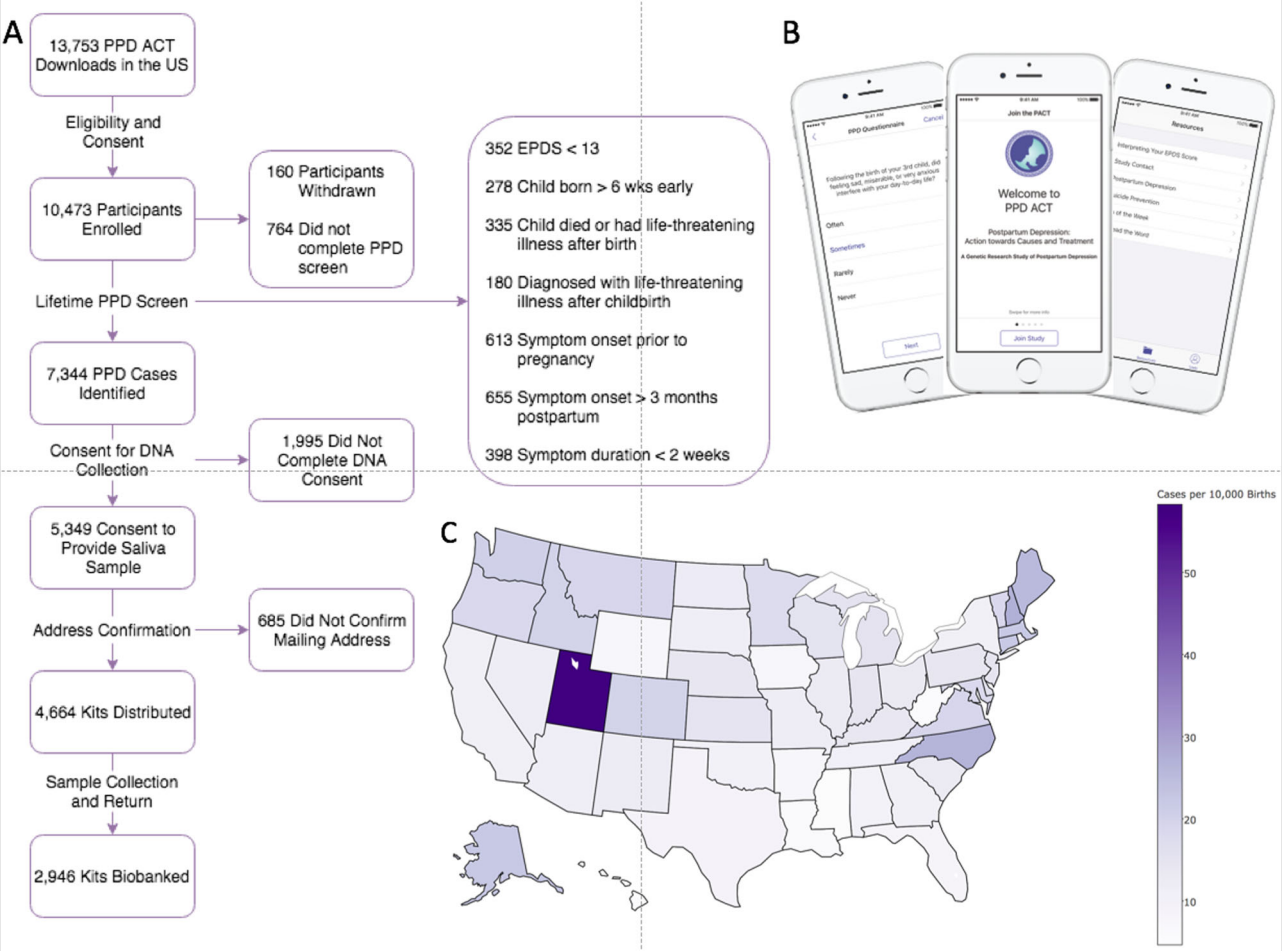
*PPD ACT* participant flow and geographic distribution in the US. **a** Flowchart of participants through the app and the number of participants that pass each step. **b** Images of *PPD ACT* app. **c** Geographic distribution of cases per 10,000 births (State-level birth rate data was taken from the National Vital Statistics Reports Final Births Report for 2015^42^)

Therefore, we developed *PPD ACT*, an Apple iOS app designed to efficiently and inexpensively assemble cases for a genetic study of PPD (Fig. 1b). We used the features of Apple’s ResearchKit platform becoming the first mobile health study focused on psychiatric genetics. Ultimately, we want to recruit 100,000 women with a lifetime history of PPD to sufficiently power GWAS. In this paper, we describe the development of *PPD ACT*, and report our experiences and results for the initial year of the study. In addition to providing a foundation for our GWAS, our aim is to provide a template for novel app-based research for other investigators, particularly those focusing on genetic sample collection.

## Results

### Study enrollment and user experience

*PPD ACT* was released to the US and Australian Apple App Stores on March 21, 2016 in partnership with the PACT Consortium (Postpartum depression: Actions towards Causes and Treatments), PPD advocacy groups, the NIMH, and the Foundation for Hope (Durham, NC). Apple provided technical assistance and several rounds of review that improved the user experience of the app.

*PPD ACT* has two basic components: participant screening for PPD and collection of DNA from PPD cases. In the US, *PPD ACT* was downloaded 13,753 times from the App Store in the year from March 21, 2016 to March 20, 2017. Fig. 1a describes the flow of users through the US version of the app.

Following download, 10,473 participants completed a basic eligibility screen (i.e., participants were female, age ≥ 18 years, fluent in English, and had had one or more births). Informed consent for PPD phenotyping followed. In psychiatric genetics research, informed consent is rarely obtained without direct interaction with a potential research subject. To comprehensively address these important issues, we engaged in extensive discussions with the UNC Institutional Review Board Committee for the Protection of Human Subjects to establish an appropriate ethical–legal framework. This included two phases of informed consent for phenotyping and DNA collection. Each consent process begins with a series of screens providing content of the consent topics followed by a short quiz to ensure comprehension. Once this is completed, participants “finger-sign” the consent document electronically and are emailed a PDF of the signed informed consent document. It is clear throughout the consent process that the participants can withdraw from the study at any time through the app or via email.

We used the lifetime version of the Edinburgh Postnatal Depression Scale (EPDS)^14^ for PPD screening. The standard EPDS^15^ is among the most commonly used and widely studied PPD screening instruments^2,16–18^ and is arguably the gold standard for PPD screening. It was developed to assess PPD and minimizes confounding of the somatic symptoms of MDD with normal infant parenting experiences (e.g., lack of sleep and tiredness)^15^. Our research group developed the modified version of the EPDS to assess lifetime history of PPD^14^ and it has been validated and used in population studies of PPD^9,19^. The EPDS-lifetime consists of 21 questions that assess symptom dimensions of the worse episode of PPD, as well as onset and duration of symptoms. Participants were scored on a scale of 0–30 with higher scores indicating greater symptom severity. We used a score of ≥13 to identify cases, a standard cutoff identified in previous population studies^15,20^. Additionally, symptom onset had to have occurred during pregnancy or in the postpartum period (<3 months postpartum) with symptom duration longer than 2 weeks. Participants who reported significant perinatal trauma (child born more than 6 weeks early, child death/life-threatening illness of infant, or maternal life-threatening illness after birth) around their worst episode of PPD were excluded due to potential confounding.

We identified 7344 women with a lifetime history of PPD. These PPD cases were invited to take part in the DNA collection portion of our study: 5349 cases completed consent for DNA collection, and 4664 confirmed their mailing address and were sent spit collection kits. After 1 year, 2946 saliva samples had been returned and biobanked at the NIMH Repository and Genomics Resource Biologic Core.

**Fig. 2:**
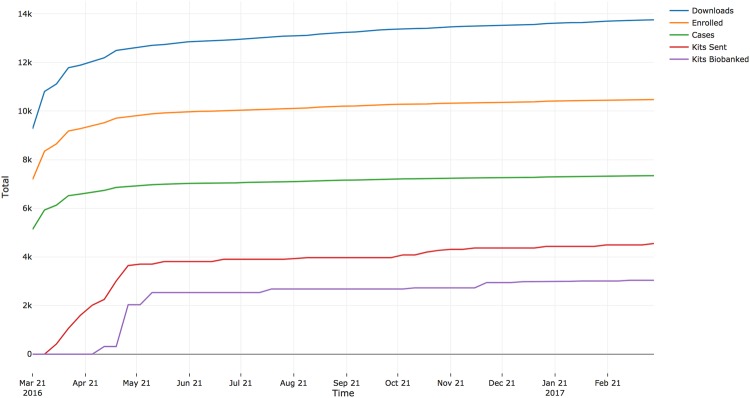
**Cumulative PPD ACT US data over time**

Figure 2 shows the cumulative totals of downloads, enrollment, cases, kits sent, and samples biobanked over the first year of the study. We obtained a 73.9% (± 8.8% standard deviation) retention in each step of participant flow, from downloading the app through returning a spit kit. The step with the highest rate of retention was the 87.2% of participants who consented to DNA collection and subsequently confirmed their mailing address. The lowest rate of retention came immediately after sending spit kits with only 63.2% of kits being returned for biobanking.

### Characteristics of PPD cases

**Fig. 3:**
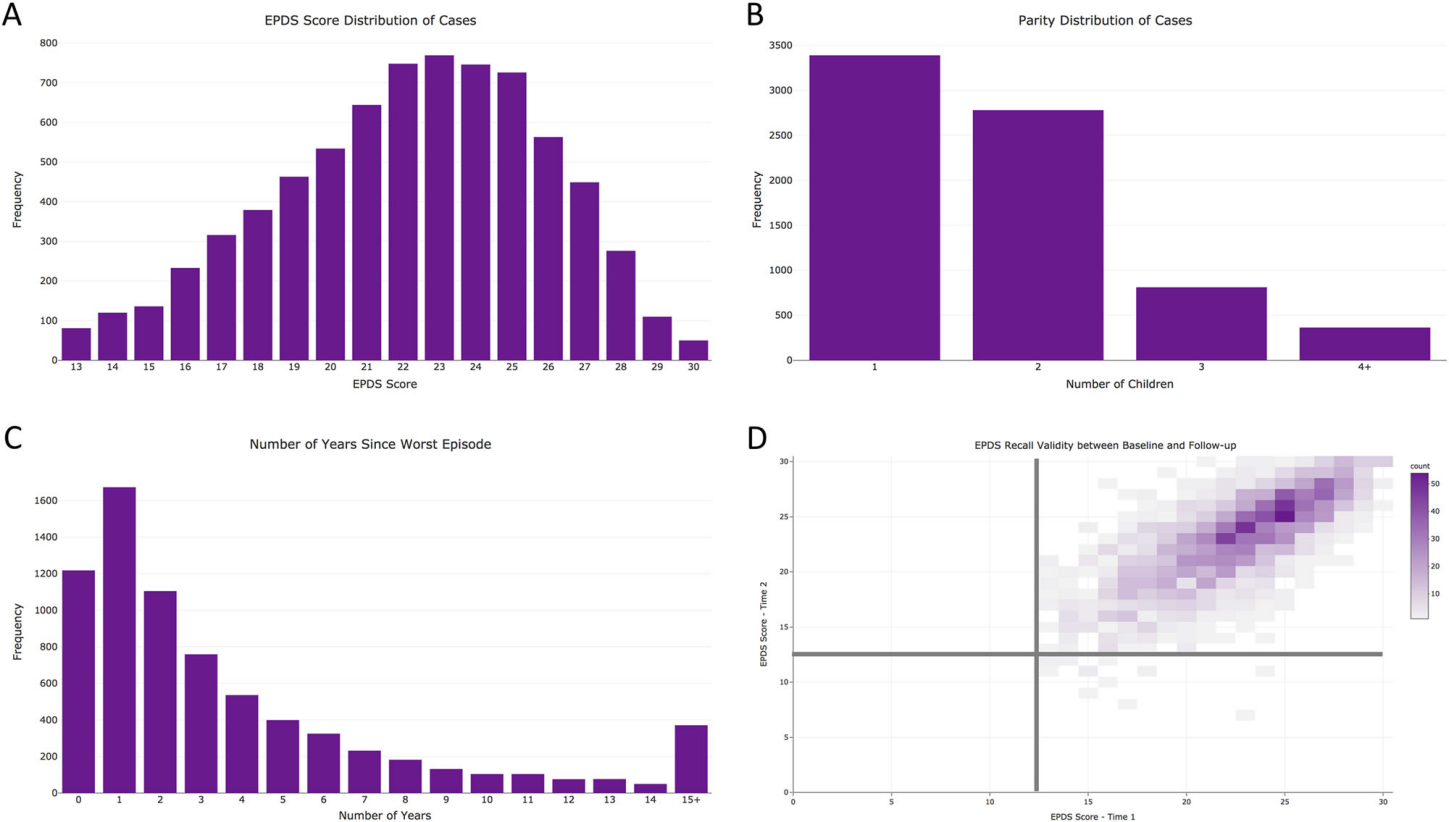
Characteristics of US ases and test–retest reliability. **a** Distribution of EPDS Scores among US cases. **b** Number of children among US cases. **c** Number of years since worst episode at the time of enrollment. **d** Correlation of EPDS scores between test and retest (>6 months after initial screening)

The 7344 cases identified via the *PPD ACT* app had a median age of 33 years (interquartile range, IQR: 29–36) and self-reported these US census race categories: 89.4% White, 5.1% Hispanic, 2.2% Asian, 1.3% Black, 0.4% Native American, 0.1% Pacific Islander, and 1.1% Other. While most cases are of a single racial group, these proportions do not significantly differ from that of the general US population (*χ*^2^(36) = 42.0; *p* = 0.23)^21^. Further, cases represent all 50 states with Utah having the highest number of cases per 10,000 births (Fig. 1c). The lifetime EPDS threshold for PPD case status was ≥13 but the median score in the cases was 23 (IQR: 20–25; Fig. 3a). This sample of PPD cases was severely affected compared to previous studies that identified PPD with high sensitivity and specificity (>80%) using threshold scores of 10–13^15,22–25^. Most cases had one birth (46.1%), followed by those with two (37.9%), three (11.0%), or four or more (5.0%) children (Fig. 3b).

**Fig. 4:**
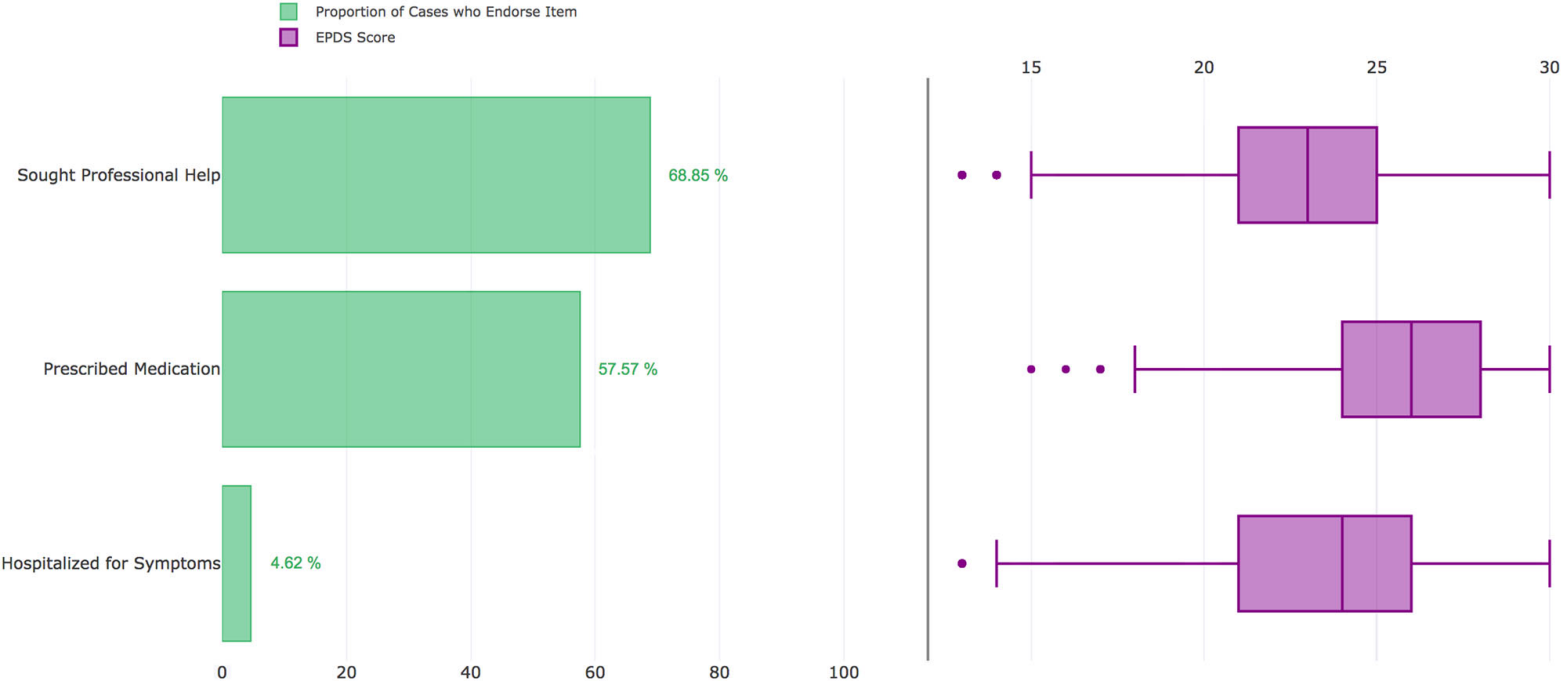
**Severity measures in US cases and respective EPDS scores**

Cases reported a median of 2 years since experiencing their worst episode of PPD (IQR: 1–5 years; Figs. 3c), and 15.8% of cases (*n* = 1,162) had given birth within 6 months of joining *PPD ACT*. Further illustrating the severity of these cases, 68.9% reported seeking professional help, 4.6% were hospitalized for PPD, and 57.6% were prescribed medications (Fig. 4). Among PPD cases who sought professional help, there was a significant increase in EPDS score (i.e., PPD severity) compared to those who did not (median 23 vs. 20; *p* = 1.63 × 10^−155^). This trend also holds true among those who were hospitalized for their symptoms (median 26 vs. 22; *p* = 3.40 × 10^−67^) and those who were prescribed medication for their symptoms (median 23 vs. 21; *p* = 1.30 × 10^−174^).

### Clinical validation of case status

To validate the ability of *PPD ACT* to identify retrospective PPD cases, we recruited women aged 18–55 years with a history of PPD from UNC Perinatal Psychiatry Outpatient Clinics. Research coordinators approached women during their scheduled appointments to explain the study. Those who agreed to participate were provided an iPod Touch to complete *PPD ACT* while in clinic. A total of 43 women with a history of psychiatrist-diagnosed lifetime PPD were recruited (median age 32 years, IQR: 26–35). The median lifetime EPDS score was 21 (IQR: 19–24), and all women were classified as cases by the app (i.e., 100% sensitivity in this opportunistic clinical sample).

### Case status recall validity

The lifetime EPDS relies on retrospective assessment of PPD which is vulnerable to recall bias. Therefore, we sought to establish the recall validity of the lifetime EPDS. Six to 9 months after enrollment, women who had met criteria for *PPD ACT* case status were sent an email requesting a second assessment of PPD using a web-based form containing the same lifetime EPDS questions that were presented previously within the app. There were 2091 PPD cases who participated in the second lifetime EPDS assessment. Fig. 3d depicts the correlation between lifetime EPDS scores taken at least 6 months apart. The median number of months between test and retest was 6.9 (IQR: 6.7–7.2). There was a high degree of agreement between assessments for which delivery preceded the worst episode of PPD (*κ* = 0.91, 95% CI: 0.91–0.91) and age of worst episode (intraclass correlation, ICC = 0.96, 95% CI: 0.96–0.97). Using the lifetime EPDS score threshold of ≥13^14^, there was 99% agreement in case status (95% CI: 0.99–1.00) among participants who completed the reassessment (Fig. 3d). Although case threshold levels of EPDS scores were reached, there was some variation in test and retest scores (ICC = 0.74, 95% CI: 0.72–0.76). Bland–Altman analysis showed a bias of −0.50 with limits of agreement from −5.8 to 4.8, meaning in 95% of cases the retest scores would be within ± 2.6 of the initial test (Supplementary Figure 1).

Using the full case definition from the initial assessment in the app (lifetime EPDS score ≥13, symptom onset during pregnancy or within 3 months postpartum, and symptom duration >2 weeks), there was 85% agreement in case status (95% CI: 0.83–0.86). This level of agreement compares favorably with that of structured clinical interviews administered by clinicians for DSM-IV defined MDD (0.61–0.80)^26–28^. Further, this 85% agreement is due to decreased agreement for time of onset (90%; 95% CI: 89–91%) and duration of worst episode (94%, 95% CI: 93–95%): women no longer being identified as cases (*n* = 314) was due to over-reporting their symptom onset during retest (initially reporting onset of 1–3 months postpartum and then changing > 3 months on follow-up assessment, *n* = 206) or under-reporting their symptom duration during retest (initially reporting symptoms lasting 2–4 weeks and the later reporting to reporting <2 weeks on follow-up, *n* = 116).

### Predictors of spit kit return

As noted above, 63.2% of consenting PPD cases who were sent a spit kit returned it. Multivariable logistic regression was used to identify putative predictors of spit kit return following address confirmation. We found that the probability of spit kit return increased with older age at enrollment (OR = 1.02; 95% CI: 1.01–1.03; *p* = 6.2 × 10^−^^6^), and decreased with greater EPDS score of worst episode (OR = 0.97; 95% CI: 0.95 – 0.99; *p* = 8.1 × 10^−04^) and longer time between address confirmation and receipt of spit kit (OR = 0.99; 95% CI: 0.99–0.99; *p* = 2.8 × 10^−37^). The latter is important as it can be controlled by study investigators.

### Implementation of *PPD ACT* in Australia

To maximize sample size for genetic studies we aim to rollout the *PPD ACT* app and research protocol in many countries around the world. Prior to the initial launch of the app, we contacted collaborators in other English speaking countries to see if we could launch simultaneously in multiple countries, and achieved this goal in Australia. However, because the Australian team wanted to use *PPD ACT* to invite participants into online MDD study which was in preparation, there was not a major Australian media campaign. Nonetheless, through global media and social media, there were >500 downloads in Australia in the first month and 604 downloads in 1 year.

**Fig. 5:**
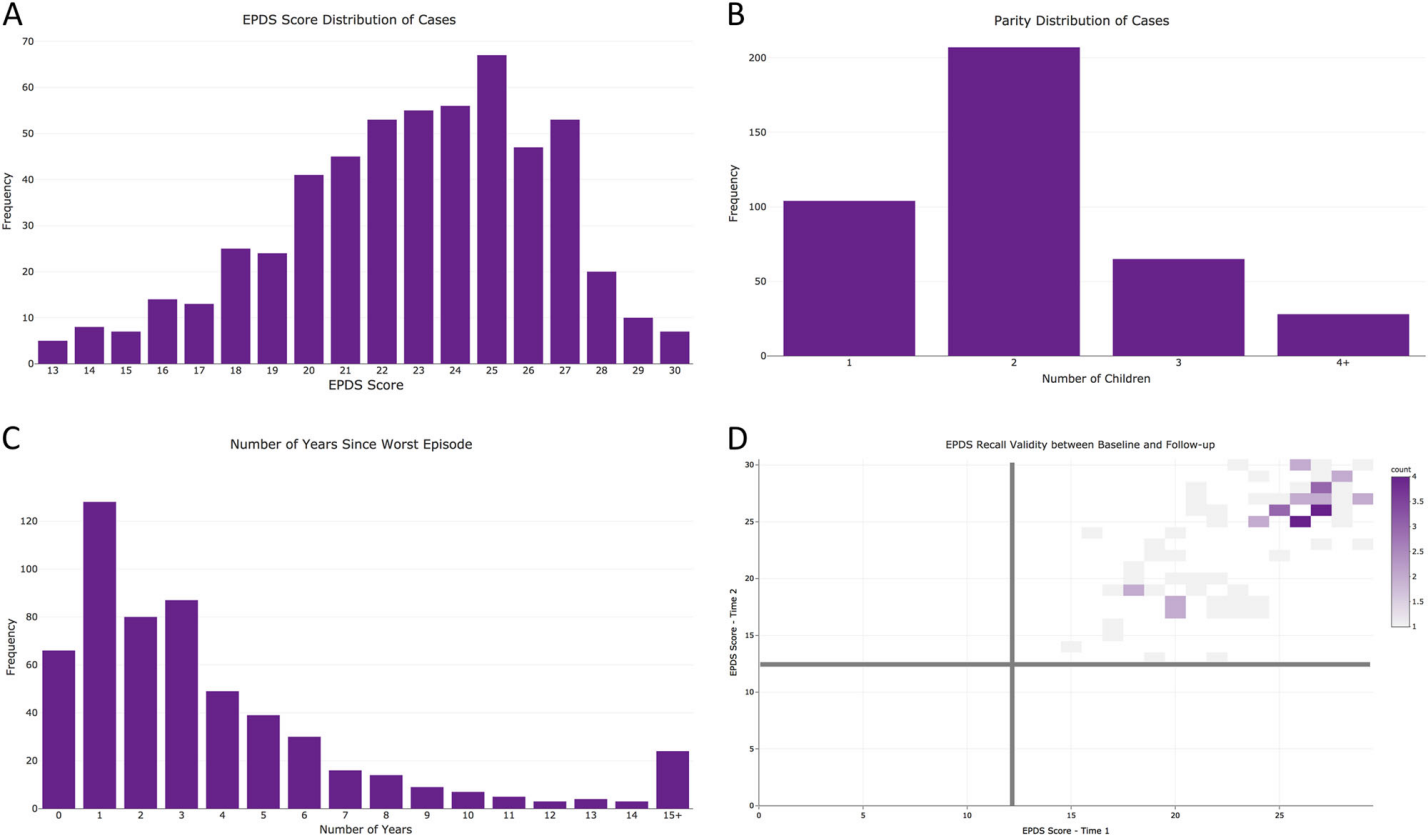
Characteristics of Australian cases and test–retest reliability. **a** Distribution of EPDS Scores among Australian cases. **b** Number of children among Australian cases. **c** Number of years since worst episode at the time of enrollment. **d** Correlation of EPDS scores between test and retest (>6 months after initial screening)

**Fig. 6:**
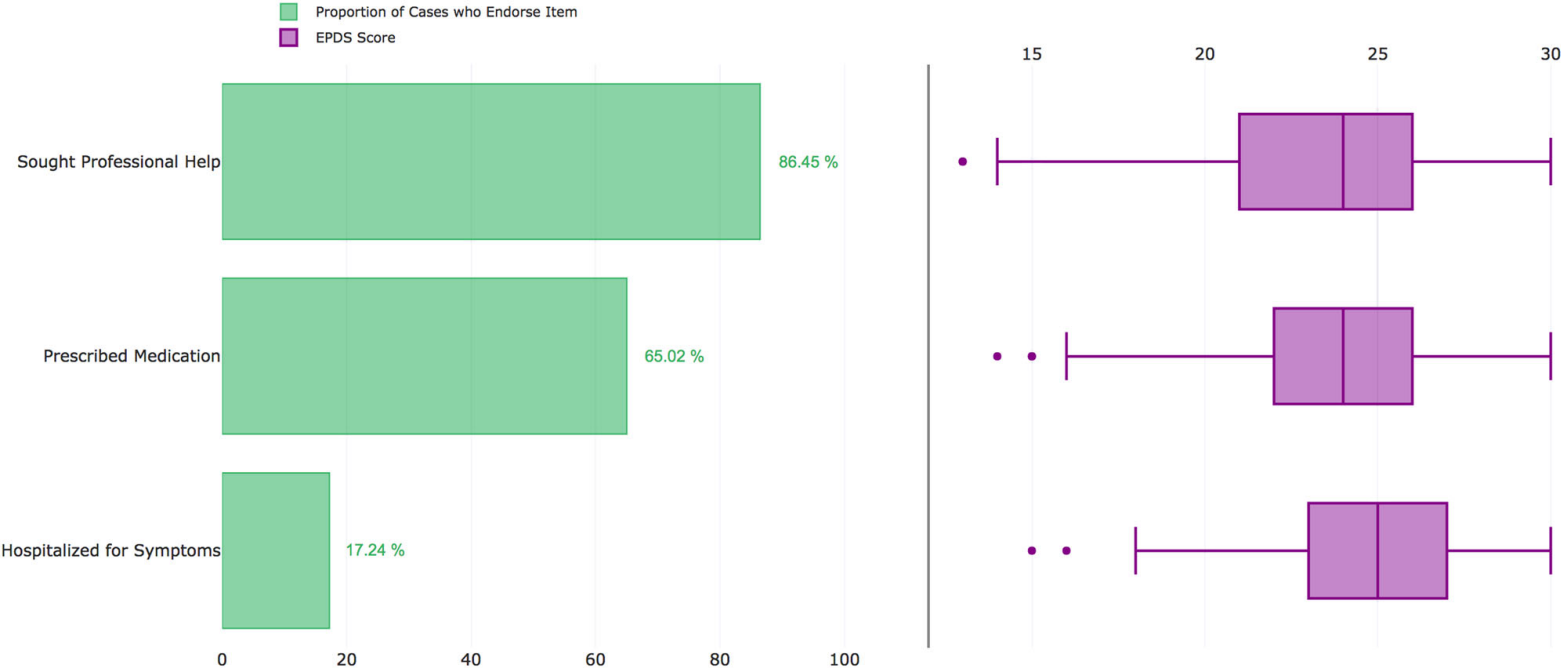
**Severity measures in Australian cases and respective EPDS scores**

A total of 411 women met the criteria for PPD (median EPDS = 23; IQR: 20–25; Fig. 5a). Among cases, 24% were primiparous, 53% had two children, 22% had three children, and 9.5% had four or more children (Fig. 5b). The median age at enrollment of cases was 33 (IQR: 30–36) and the median age at onset of PPD was 30 (IQR: 26–32). The average time since experiencing PPD was 4 years with a median of 3 years (IQR: 1–5, Fig. 5c). Rates of help seeking (86.3%), medication use (65.9%), and hospitalization (17.3%) were higher than in the US sample, which may reflect differences in healthcare between the two countries (Fig. 6). Visits to general practitioners (GPs) and specialists, and prescription medicines are subsidized by the Australian government, meaning women may have been more likely to seek help. Women reporting having been sought professional help had higher EPDS scores (median 24 vs. 20; *p* = 1.9 × 10^−8^). Likewise, those reporting having been prescribed medication reported worse symptom severity than those who did not (median 25 vs 22; *p* = 4.1 × 10^−11^) and those who reported being hospitalized had the worst symptoms (median 25 vs. 23; *p* = 1.5 × 10^−6^).

Women who completed the EPDS in the app were sent a follow-up email to say that funding for DNA collection is dependent upon completion of a more comprehensive questionnaire that assesses lifetime history of mental illness and use of antidepressant medication. Most participants were invited into the pilot testing phase in September 2016, with the full study launched in April 2017. Acknowledging the significant delay between the launch of the app and invitation to the online questionnaire, 140 women enrolled in the online study, of whom 106 were asked to provide a DNA sample and 100 have returned a spit kit. The questionnaire has a modular form with the first module being compulsory. Optional modules ask questions about pregnancy and PPD, including the EPDS. To date, 71 women have completed this module, thus providing test–retest reliability data for the EPDS. All women who completed the online reassessment scored greater than case threshold of ≥13^14^ (Fig. 5d). The correlation between EPDS scores from the app and the online questionnaire was 0.69 (ICC = 0.69, 95% CI: 0.54–0.79). Bland–Altman analysis showed a bias of −0.32 with limits of agreement from −6.8 to 6.1, meaning in 95% of Australian cases the retest scores would be within ±3.2 of the initial test (Supplementary Figure 2). Further, 92% of the women who met the criteria for having had PPD from the app also met the criteria in the online questionnaire. All of the women who did not meet criteria for PPD in the online questionnaire reported symptoms starting more than 3 months after childbirth. There was also substantial agreement for time of onset (91%, 95% CI: 82–97%) and duration of worst episode (100%, 95% CI: 95–100%).

## Discussion

To our knowledge, *PPD ACT* is the first mobile health application for a psychiatric genetics study designed to screen and collect samples directly from participants. Using Apple ResearchKit, we were able to identify more than 7300 PPD cases and biobank nearly 3000 genetic samples during the first year of recruitment. The cases identified suffered severe lifetime episodes of PPD (median EPDS = 23; IQR: 20–25). In addition, we showed that our assessments of PPD have highlevels of agreement with diagnosis made by a skilled provider in a clinical setting and high test–retest reliability.

PPD is a common and debilitating disorder that affects new mothers and infants for the duration of the depressive episode and can have long-lasting negative outcomes for the child. There are nearly four million births per year in the US. 40% of births are to first-time mothers who usually do not know their own risk for PPD. More than 500,000 women each year will suffer with PPD, and many of these women with PPD will go undiagnosed and untreated^29^. Given the pervasive use of smartphones (75% of all US women), we believed that *PPD ACT* would allow us to reach a large population of women who had suffered with PPD (current or lifetime) and who would be interested in sharing their experience using a mobile health application and contribute saliva samples to a research study focused on understanding the genetic signature of PPD. We were able to use ResearchKit to bring PPD research to a much larger population compared to traditional studies that require participants to visit specific research centers. Importantly, our partnership with advocacy groups that helped us spread the word and encourage participation of women with PPD in this study was vital to the success of our recruitment and sample collection.

Our highest rate of enrollment came within the first month following launch, identifying 204.9 cases per day, compared to 2.4 cases per day in the subsequent 11 months (Fig. 2). Characteristics of PPD (severe symptoms, relatively common, often understudied) likely contributed to the success of this study compared to other mobile health initiatives (i.e. asthma^30^, skin cancer^31^, Parkinson^32^). Additionally, lower demands on the participant with respect to data collection (questionnaire + sample kit/mail) may have added to our overall success in recruitment. We attribute our initial spike in participation to the large media campaign at launch, which we coordinated with Apple, national media (the *New York Times*, CNN), postpartum depression advocacy groups (Postpartum Progress and Postpartum Support International), and our US and Australian research teams. As rates of enrollment began to decrease, we regularly released social media and advertising content, which resulted in consistent, yet modest increases in enrollment. We found that constant media presence is needed to keep engagement with potential participants.

Overall, we enrolled a population of cases that experienced severe episodes of PPD (median EPDS = 23). A large proportion of cases reported symptoms so severe they sought professional help or were prescribed medication, which corresponded with significantly increased symptom severity compared to other cases (Figs. 4 and 6). This clinically meaningful level of suffering likely increased motivation for women that had suffered with PPD to participate in our study. Further, the clinical validation aspect of our project gives us strong confidence in our ability to identify PPD cases, along with reassessment of participants with the EPDS 6–9 months after initial enrollment. Importantly, the high agreement in case determination (85%; 95% CI: 0.83–0.86) was greater than clinicians’ agreement of DSM-IV defined MDD (0.61–0.80)^26–28^. Using the lifetime version of the EPDS also allowed us to accurately determine reliability of which childbirth proceeded worst episode (*κ* = 0.91), age at worst episode (ICC = 0.96), onset (90% agreement), and duration of worst episode (94% agreement) in a cohort larger than any studied previously^33,34^.

The differences in responses between the initial assessment of the lifetime EPDS on the app and the follow-up assessment 6–9 months later could be due to the time between test and retest, although this was not important empirically (*r*^2^ = 0.01). However, the lack of agreement between women discordant for case status upon reassessment may be due to small changes in responses for onset and duration between test and retest. These small changes in response, such as initially reporting onset of symptoms at 1–3 months postpartum and then subsequently reporting onset > 3 months postpartum upon reassessment, could indicate these women fell at a cutoff for case definition (i.e. 3 months for true onset), but recall bias affected participant responses. Recall bias is expected when relying on retrospective assessments. Our test–retest reliability of lifetime EPDS scores is the first in the literature. Compared to previously reported test–retest reliability for the standard EPDS^34^, we report on a larger sample size (2091 in our study v. 118) and increased time between test and retest (209 days in our study v. 2.8). Therefore, we are more confident in the test–retest reliability of lifetime EPDS scores (ICC = 0.74) despite being lower than previously reported for the standard EPDS (ICC = 0.92)^34^.

### Lessons learned

The major strength of this study was our ability to recruit 7344 cases in the US with PPD and 411 in Australia over the course of a year, all at a fraction of the cost of traditional genetic sample recruitment. To date, this is the most rapidly conducted study of PPD and is nearly half of the cases analyzed in the first GWAS of MDD performed by the PGC in 2013^35^. There were many collaborations required to provide the diverse expertise needed to successfully complete this research study. For example, a constant dialog with the UNC IRB regarding the protection of participants was critical to the successful completion of *PPD ACT*. As geneticists and clinicians, we required an experienced app development team, design input from Apple, information technology support from the UNC Office of Information Systems, marketing assistance for public relations and media outreach, input from consumers and advocacy groups, and our scientific advisory board to ensure we maintained research rigor and designed a usable, responsive product as *PPD ACT* moved from one development stage to the next. This rigorous approach was also illustrated in our clinical and recall validation studies, which ensured we captured true PPD cases.

Additionally, we have created a research platform that can be deployed on an international scale. Our initial development included the ability to customize *PPD ACT* based on a country’s cultural, legal, or ethical considerations. This includes full language translation. Our deployment into Australia was rapid (3 months prior to launch) and included text customization to reflect Australian preferred wording for ease of understanding and legal protections specific to Australia (see Methods). The qualitative consistency of data collection across the US and Australian sights is notable (see Figures).

Another notable strength of this study is that many women subsequently sought help based on the feedback received from the app on their symptom severity and the built-in features to find local resources for care. We received many emails from providers across the U.S. that they were seeing new patients who had used the app to screen for PPD. While this was not the primary goal of this study, it is a positive and clinically meaningful outcome.

However, as a first in the field, we recognize there are improvements that can be made. First, there are many points in our user experience pipeline where participants were lost. Our average retention of ~ 75% can be improved, particularly in the steps following case identification (DNA collection consent through spit kit return). Investigators should send spit kits to consenting cases as quickly as possible. We also learned that continuous marketing of the app and consistent engagement of study participants drive the success of our study. This is highlighted by the fact that enrollment was highest when the media coverage was highest immediately following the launch of *PPD ACT*. The enrollment numbers also rose following advertising releases on social media. Once participants consented to provide a DNA sample, ensuring kits were mailed in a timely manner to capitalize on participant interest. This was shown with the latency from address confirmation to kits being mailed being a significant predictor of spit kit return. However, our return rate for spit collection kits (63.2%) is higher than many other previously reported studies collecting saliva samples via post (42.2–58.5%)^36–38^. Only a single study reported a higher return rate of 85% in a population of elderly individuals^39^, which supports our finding of increased-age increasing rate of return.

### Future directions

This population of women who experienced PPD has been highly motivated to share their experiences with PPD, to find causes and prevent future suffering. We have received many messages of gratitude and thanks from study participants for our work on an often neglected disorder. Our goal is to integrate this outpouring of support for PPD research into our ongoing efforts to expand the current iteration of *PPD ACT* to collect even more data and better characterize the trajectories of women who develop PPD. Specifically, the cases we have recruited are largely of European ancestry, though these proportions are not statistically different from those of the U.S. population. A primary aim moving forward is to increase the diversity of our participants. Therefore, on April 27, 2017, we released an Android version of *PPD ACT*, which allows us to capture a larger and more complete population of women who experienced PPD. We hope to double our recruitment numbers with this expansion since Android has a greater market share than iOS. We also recently released (October 5, 2017) a Spanish-language version of *PPD ACT* in the US for both iOS and Android. Additionally, we are expanding into new countries and launched *PPD ACT* in Canada on April 27, 2017, with plans to release in Denmark and the UK next. We are in planning discussion with many other countries (Brazil, Germany, Israel, Spain, Sweden). Further, in the next phase, we aim to add a treatment component to aid women who are actively experiencing PPD symptoms.

In conclusion, we know from previous genetic studies of psychiatric disorders (i.e. MDD^35^, schizophrenia^40^) that it takes many tens of thousands of cases to identify a genetic signal. It will take thousands of women at many international sites to participate in *PPD ACT* to gain a better understanding of the genetic architecture of PPD. However, the response we obtained in just the first year of our study makes us hopeful that we are well on the way to understanding the genetic signature of postpartum mood disorders, thereby having critical knowledge that will improve detection, prevention, and treatment of these often devastating conditions.

## Online methods

### Study design

The *PPD ACT* study has two basic components: participant screening for PPD and collection of DNA from PPD cases. Fig. 1 illustrates the participant flow through each part of the study. Once a woman who believes she might have had PPD downloads the app, she completes a basic eligibility quiz (female, age ≥ 18, English speaking, number of births ≥1). Eligible women are presented with informed consent for participation in screening. The full informed consent document is presented through a series of screens followed by a quiz to ensure the participant fully understands the content. If the participant consents, she signs the consent document with her finger on the device and the full, signed copy of the document is emailed to her.

Upon completion of the screening informed consent, participants are screened for a lifetime history of PPD. If deemed to be a case, they are invited to participate in DNA collection. The user is presented with a second informed consent specific to DNA collection, followed by another quiz to demonstrate understanding. Women then finger-sign the DNA informed consent document and are emailed a full, signed copy of the document. After this second consent, the user provides their mailing address, which they must then validate via email prior to a DNA collection kit (saliva) being mailed to them. Once the participant provides a saliva sample, they use the provided packaging and postage to send the samples directly to the NIMH Repository and Genomics Resource (NIMH-RGR) Biologic Core (Rutgers University’s RUCDR Infinite Biologics).

### Development of app storyboard

We performed a comprehensive literature search to identify which PPD screening instruments were best suited for adaptation into a mobile application. Given that our primary aim was to conduct a genetic study, our assessment for PPD needed to capture lifetime PPD. Some years earlier, we had developed and established a lifetime version of the EPDS^14^ and used this as the backbone for our PPD screen. The standard EPDS^15^ was created to assess current symptoms of PPD. We used the lifetime EPDS in a large-scale twin survey in Sweden that showed the heritability of PPD to be 54%^9^. Additional questions about perinatal trauma were developed by PACT Consortium experts: Professors Veerle Bergink (Erasmus Medical Centre, The Netherlands), Trine Munk-Olsen (Aarhus University, Denmark), and Emma Robertson-Blackmore (Halifax Health, USA). Additional standardized questions were taken from the NIH PhenX Toolkit^41^.

To maximize efficiency and to minimize developer costs, we developed a “storyboard” (or “wireframe”) prior to involvement of an app developer. This storyboard contained the text and graphical content of every screen including all questions and responses, and clear instructions for every screen touch. It captured the full “logic” of the app, complete with proposed layouts and content for each screen, and logic between screens and questions. All content was compliant with the Apple ResearchKit framework. We chose to implement our study using the open-source ResearchKit platform because it offered standard templates and formats for many elements of our study (i.e., introduction, eligibility screening, consent and quiz, and survey administration). We performed a comprehensive check of logic within the storyboard, testing all combination of responses to ensure all possible options resulted in desired endpoints.

The full storyboard was vetted by our scientific advisory board: Professors Naomi Wray (University of Queensland, Australia), Douglas Levinson (Stanford University, USA), Brenda Penninx (VU University Medical Center, Netherlands), and Cathryn Lewis (King’s College London, UK), who are experts in MDD assessment, epidemiology, and genetics. Feedback from the scientific advisory board was integrated into the storyboard with changes to logic triggering another full evaluation of all app logic (testing all combination of responses).

Following several rounds of review, a final version of the storyboard was completed. This storyboard was used to generate comprehensive quotes for the app development from potential software developers during the bidding process.

### *PPD ACT* app development and data security

*PPD ACT* version 1.0.0 was built by Little Green Software (LGS; Durham, NC; www.littlegreensoftware.com) using Apple’s ResearchKit following the specifications defined by the storyboard. We collaborated with Foundation of Hope (Raleigh, NC; www.walkforhope.com), the National Institute of Mental Health (NIMH; www.nimh.nih.gov), Postpartum Progress, and Apple (Cupertino, CA; www.apple.com) to develop and launch the PPD ACT App Study on March 21, 2016. This study was approved by the UNC Institutional Review Board Committee for the Protection of Human Subjects.

We chose to first pursue the iOS version of *PPD ACT* to take advantage of the pre-built framework developed by Apple. ResearchKit provided the starting point for the user interface/design. Features of the user experience included study introduction layout screens, informed consent presentation and quiz, along with standardized input types for questionnaires (i.e. wheel selection for multiple choice questions). However, ResearchKit did not provide any mechanism to securely send and store user information to a server. For this, LGS designed a custom database on UNC servers and an interface (RESTful API using IBM StrongLoop LoopBack Framework) for *PPD ACT* to securely transmit all research data.

LGS worked closely with the UNC Office of Information Systems to deploy the server and store the study data in a fully encrypted Oracle SQL database, following the best practice of encryption-at-rest. The encrypted database is accessible only from the server by the server application and designated IT administrative staff. LGS and UNC IT performed rigorous load testing of servers and their interfaces to ensure it could handle high usage periods.

Researchers access study data through a password-protected administrator’s web-based portal. The portal uses public/private key encryption to securely transmit authorized study data to the researcher. By encrypting the data with the researcher’s public key, he or she is the only one able to decrypt and view it.

### Finalizing the app

UNC and LGS performed thorough quality assurance testing at the end of each major development milestone. This included testing of all logic within the app to ensure a match to the storyboard, uniform presentation of all content, and accurate case determination. Upon creation of a stable staging build, we held focus groups with women using test versions of the app at the UNC Women’s Hospital and with members of Postpartum Progress to gain insight and valuable feedback from potential participants. Feedback was integrated into app design, which was followed by another cycle of focus group testing and feedback integration. In addition, our preliminary version of the app was distributed to members of our scientific advisory board for vetting. This review by our scientific advisory board not only included a meticulous check of all PPD assessments but also included real-world usage of the app (i.e. completion of the app on public transportation with variable network connection).

We worked closely with Apple while developing the *PPD ACT* app to ensure it followed the overall ResearchKit guidelines and met the level of quality for ResearchKit apps. Apple user experience teams provided input on the overall user experience and design elements LGS added to the ResearchKit core functionality. This feedback included changes to logic to improve user flow through the app, breaking longer questions into more concise questions, and formatting text for easier comprehension. Following review by Apple, feedback was incorporated into the app prior to release to the App Store.

### Informed consent procedures

In psychiatric genetics research studies, informed consent had rarely been obtained solely within an app or website but was required for this study. The research team held extensive discussions with the UNC Institutional Review Board Committee for the Protection of Human Subjects to establish an appropriate ethical–legal framework.

The UNC IRB requested that we include two separate informed consents, one for each part of the study (screening for PPD and participation in DNA collection). For each consent, the content of the IRB approved consent form is displayed through a series of screens explaining the major points of informed consent, followed by a quiz to assess comprehension of the content. Finally, the app obtains the participant’s signature for an executed consent document. A signed copy of the informed consent document is immediately emailed to the participant as a PDF file once completed. Another copy of the signed informed consent document is stored on the UNC servers.

Discussions with the UNC IRB and the Office of Human Research Ethics ensured careful preparation for all consent procedures for our novel study. There were few challenges in developing the screening informed consent document since it closely resembled standard studies that use research surveys. The unique issues to consider included secure storage of data on a mobile device (iPhone or iPad) and ensuring participants fully understood what was being asked of them in the consent document. To address data protection on the device, we ensured no data was permanently stored on the device. Encrypted data was only temporarily stored on the device until pushed to the UNC servers. To address concerns over participant understanding of proposed participation, ResearchKit’s consent screens and quiz ensure that participants demonstrate a full understanding prior to enrolling in the study. The entirety of the informed consent document is presented to the user through several screens. Content is divided into smaller, more comprehensive chunks based on related content (data security, privacy, data use, time commitment, study content, withdrawing, re-contact). A consent quiz requiring all questions are answered correctly is administered prior to signing the consent document. This ensures the participant understands what is involved when participating in our research study.

More substantive concerns were raised when developing the consent to collect DNA including: (1) how participants can withdraw from the study, (2) what happens to participant DNA if they withdraw from the study, (3) identifying all of the risks and benefits associated with providing a DNA sample, and (4) effectively describing how participant DNA will be used. These points were essential to clarify, especially since we were the first study at UNC to collect samples for a genetic study using an app rather than an in-person collection. Participants can withdraw from the study by emailing UNC researchers or within the app. Upon withdrawal, participant DNA and health information is removed from all study repositories. Participants understand that we would use their DNA to “measure 500,000 or more genetic markers (or “signposts”) scattered across the genome… We would carefully analyze these DNA measurements to find the places in the genome that differ in women with PPD or PPP.” The risks for participating in the *PPD ACT* study were similar to other genetic research studies, but emphasis is placed on these risks so that the participant fully comprehends what providing a DNA sample entails. We describe that, although unlikely, it is possible that unauthorized individuals may gain access to samples or genetic data, which may be traced to protected health information. While the risk of this occurring is very small, it may lead to discrimination based on the contents of your genome. To mitigate this, we detail the United States law called the Genetic Information Nondiscrimination Act which makes it illegal for health insurance companies, group health plans, and most employers to discriminate on the basis of genetic information. Additionally, we make participants aware that some researchers with access to their genetic data may have financial interests in studying DNA with no plans to provide any compensation to participants or their heirs.

### Customizing the *PPD ACT* app for use in Australia

Here, we detail the changes in *PPD ACT* needed for the Australian version based on cultural differences and conversations with the Bellberry Ethics Committee. In general, the wording needed to be more polite, less directive, and phrased to ensure that the participant felt in control of their participation. For example, we changed “In this study, we will get a lot of information about you.” to “In this study, with your consent, we will collect information about you.” Some other examples of changed words were “legal mandate”, “subjects”, “sponsor” to “legal requirement”, “participants”, and “funding”, respectively.

Other changes directly reflected the local governance and ethical requirements under the Australian National Health and Medical Research Council National Statement on Ethical Conduct in Human Research 2007. For example, the term “genetic test” is nuanced to mean a diagnostic test, hence all references to “genetic tests” and genetic results” were changed to “genetic screening” and “genetic information”. Additionally, the resources listed for online help were updated to those based in Australia. The US *PPD ACT* app includes a screen that asks “Have you had thoughts of harming yourself in the past month? Often, Sometimes, Rarely, Never.” The inclusion of this question in the Australian version would have prompted the need to include an adverse event plan, requiring 24-hour monitoring of responses and active intervention for participants endorsing the question. Endorsement followed by suggestions to contact a general practitioner or mental health provider is not considered sufficient duty of care toward participants. To alleviate the administrative burden implied by the adverse events plan it was decided to exclude this question.

Finally, the most substantive change was about the collection of a biological sample, because the Australian team did not have specific funding to fund the spit kit and so the consenting process was altered to advise participants of this. Although participants were consented for the collection of a biological sample, we advised participants we would recontact them to continue their participation either as part of this project or another project. We did this, enrolling them through the consent process of our online MDD project (ongoing).

### Planning a media campaign for the launch of the *PPD ACT* app

*PPD ACT* was made available on the U.S. and Australian App Stores on March 21, 2016. An iOS device (i.e. iPhone, iPad, iPod Touch) running iOS 8 or later was required to download and use the app. The largest recruitment boon occurred during the app launch with concurrent press releases by UNC, Apple, and Postpartum Progress. *PPD ACT* was also featured in an article by the New York Times published on the day of the launch. In addition, we maximized our online presence to increase exposure to potential participants. Social media accounts (i.e. Twitter and Facebook) were created along with an updated website. UNC Public Affairs and Marketing and Capstrat (Raleigh, NC) assisted by placing social media advertisements at strategic intervals to help keep the number of participants steady over time. Additionally, our goal was to prevent errors that would yield bad publicity, specifically by actively monitoring social media and App Store feedback to answer any questions and solve problems.

### Participant eligibility criteria

Basic eligibility to participate in screening for PPD requires: female sex, age ≥ 18 years, ≥1 live birth, and belief of having experienced a lifetime episode of PPD. Cases of lifetime PPD are defined as having an EPDS total score ≥ 13, symptom onset during pregnancy or in postpartum period (<3 months postpartum), symptom duration > 2 weeks, but with no report of a child born more than 6 weeks early, death of infant or life-threatening illness after birth, or maternal life-threatening illness after birth. Women who have been pregnant without a live childbirth, non-English speakers, and those who could not document understanding of the consent based on the comprehension quizzes are ineligible for the study.

### Address confirmation, spit kit collection, and biobanking

Following consent to participate in saliva sample collection, cases are asked to confirm their mailing address using a web-based form sent by email. Spit kits are provided by the NIMH Repository and Genomics Resource and mailed from UNC upon address confirmation. Kits are barcoded with each being associated with a single participant. Once participants have provided their sample, they mail them, at no cost to the participant, to the NIMH Repository, where samples are inventoried and stored at −80 °C. The repository only receives the participant study ID and kit barcode, both of which are de-identified. All protected health information is stored securely at UNC.

### Clinical validation in UNC hospitals

For clinical validation, women aged 18–55 years with a history of PPD were recruited from UNC Perinatal Psychiatry Outpatient Clinic and OB-GYN. Research coordinators approached women during their scheduled appointments to explain the study. Those who agreed to participate were given an iPod Touch to complete *PPD ACT* while in clinic. Research coordinators recorded the app calculated EPDS score, the time it took to complete participation, and any feedback participants had on user experience. Clinician diagnosis of PPD was recorded from participant medical records.

### Recall validity with second EPDS assessment

Six to 9 months following initial enrollment, women who met criteria for case status initially were sent an email requesting a second assessment of PPD using a web-based form. This form contained the same lifetime EPDS questions that were presented previously within the app. For reassessment, cases were defined as having an EPDS total score ≥ 13, symptom onset during pregnancy or in the postpartum period (<3 months postpartum), and symptom duration greater than 2 weeks.

### Data analysis

Analyses were conducted using R (v3.2.2). Descriptive statistics are reported using percentages for categorical variables and medians with (IQRs) for continuous variables. State-level birth rate data were taken from the National Vital Statistics Reports Final Births Report for 2015^42^. Intraclass correlation coefficients (ICC) were used to measure test–retest reliability for continuous variables, using the *irr* package (v0.84). Binomial tests were used to measure agreement for binary variables. Squared weighted Cohen’s kappas were used to measure test–retest reliability for categorical variables, using the *rel* package (v.1.3.0).

## Electronic supplementary material

Supplemental Figure 1

Supplemental Figure 2
