# Supplementary figures and images for: PPD ACT: an app-based genetic study of postpartum depression
Source: Transl Psychiatry. 2018 Nov 29;8:260. doi: 10.1038/s41398-018-0305-5 (PMC6265256; doi:10.1038/s41398-018-0305-5)

# Bland-Altman Plot of US EPDS Scores

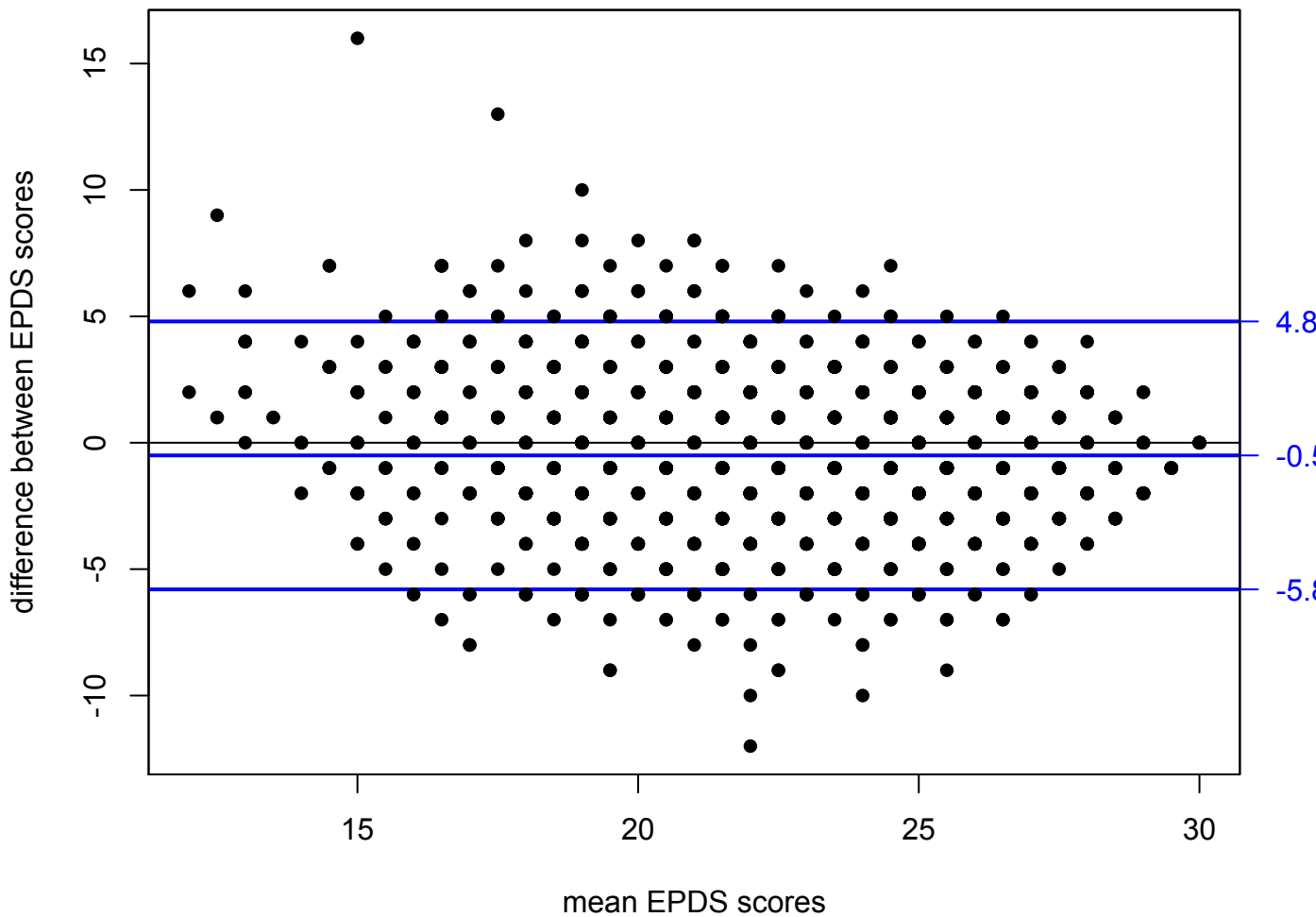

Supplement: Supplementary file 1 — Supplemental Figure 1 [file 41398_2018_305_MOESM1_ESM.pdf]

**Bland-Altman Plot of Australian EPDS Scores**

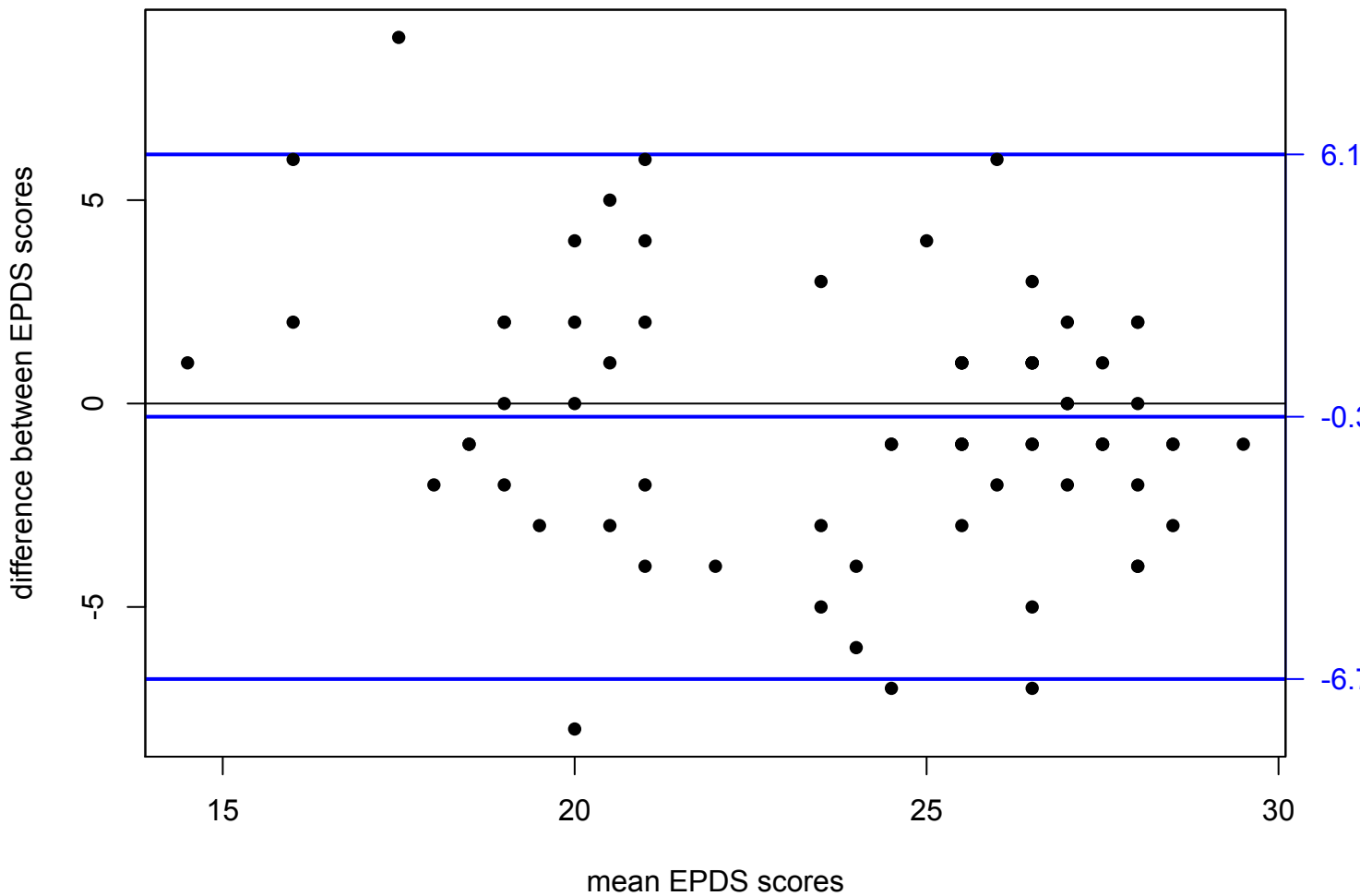

Supplement: Supplementary file 2 — Supplemental Figure 2 [file 41398_2018_305_MOESM2_ESM.pdf]
